# Supramaximal Resection in Glioblastoma: Expanding Surgical Boundaries in the Era of Precision Neuro-Oncology—A Systematic Review

**DOI:** 10.3390/cancers18071182

**Published:** 2026-04-07

**Authors:** Stuart D. Harper, Travis Perryman, Brandon Carlson-Clarke, Shivani Baisiwala, Brandon Rogowski, Amani Carson, Isha Sharma, Shail G. Patel, Eliana S. Oduro, Alondra Delgadillo, Nishvith Sudhakar, Mahmoud I. Youssef, Kunal S. Patel

**Affiliations:** 1 Department of Neurosurgery, David Geffen School of Medicine, University of California Los Angeles, Los Angeles, CA 90095, USA; sdharper@mednet.ucla.edu (S.D.H.); tperryman@mednet.ucla.edu (T.P.); bcarlsonclarke@mednet.ucla.edu (B.C.-C.); sbaisiwala@mednet.ucla.edu (S.B.); brogowski@mednet.ucla.edu (B.R.); amanicarson@mednet.ucla.edu (A.C.); ishasharma@mednet.ucla.edu (I.S.); sgpatel@mednet.ucla.edu (S.G.P.); esoduro@g.ucla.edu (E.S.O.); alondraucla@g.ucla.edu (A.D.); nsudhakar0@ivc.edu (N.S.); myoussef@mednet.ucla.edu (M.I.Y.); 2 Jonsson Comprehensive Cancer Center, University of California Los Angeles, Los Angeles, CA 90095, USA

**Keywords:** glioblastoma, supramaximal resection, extent of resection, non-contrast-enhancing tumor, FLAIR resection, awake craniotomy, intraoperative imaging, functional mapping, tumor infiltration, systematic review

## Abstract

**Background:** Glioblastoma remains the most aggressive and treatment-resistant primary brain tumor, with patient outcomes strongly associated with the extent of surgical resection. Tumor recurrence is largely driven by infiltrating glioma cells that extend beyond the contrast-enhancing margin, which has traditionally served as the boundary for surgical resection. Advances in pre- and intraoperative imaging, functional mapping, and fluorescence guidance have challenged the conventional definition of “maximal safe resection” and given rise to the concept of supramaximal resection (SMR). This technique, where surgical resection extends beyond the contrast-enhancing border, has garnered significant interest in recent years and shown promising preliminary survival outcomes. However, the lack of standardized definitions and methodological consistency has limited reproducibility and clinical adoption. **Methods:** A systematic literature search of PubMed/MEDLINE, Embase, and Web of Science was performed from database inception through March 2026 in accordance with PRISMA guidelines. Studies investigating resection beyond the contrast-enhancing tumor margin in adult glioblastoma patients were evaluated for inclusion. **Results:** A total of 1045 records were identified, with 37 studies meeting inclusion criteria. Across studies, SMR was frequently associated with improved progression-free and overall survival in selected patients, particularly following complete contrast-enhancing tumor resection. However, substantial heterogeneity exists in SMR definitions, and the current body of evidence is largely retrospective and derived from high-volume centers. **Conclusions:** SMR represents a promising extension of maximal safe resection targeting infiltrative tumor beyond conventional imaging boundaries. While emerging evidence suggests survival benefits, variability in methodology and patient-specific factors require cautious interpretation. Future standardization and prospective validation are needed to better define the role of SMR within multimodal glioblastoma treatment.

## Simple Summary

Glioblastoma is characterized by diffuse tumor cell infiltration, which extends beyond the contrast-enhancing margin visible on standard imaging and contributes to early local recurrence after surgery. Supramaximal resection seeks to extend surgical boundaries into non-contrast-enhancing regions to reduce residual infiltrative disease, but its definition and clinical implementation remain highly variable. This systematic review synthesizes the biological rationale, clinical evidence, and emerging surgical and imaging technologies supporting supramaximal resection, while highlighting current limitations and the need for standardized frameworks to rigorously evaluate this evolving surgical strategy.

## 1. Introduction

Glioblastoma remains the most aggressive primary malignant brain tumor in adults, with a median overall survival of 12–15 months [1,2,3]. The currently accepted standard-of-care management involves maximal safe surgical resection of the contrast-enhancing (CE) tumor, followed by adjuvant chemoradiation [4]. While incremental advances in systemic and radiation therapies have modestly improved outcomes over recent decades [5,6], glioblastoma continues to carry a near-universally fatal course characterized by disease recurrence and progression.

Extent of resection (EOR) is one of the strongest modifiable prognostic factors in glioblastoma care, with numerous studies demonstrating improved survival with more complete resection of CE tumor [7,8,9,10]. However, the traditional goal of “gross total resection” is inherently constrained by the limitations of conventional magnetic resonance imaging (MRI) studies. Glioblastoma cells are known to infiltrate far beyond the CE boundary into non-contrast-enhancing (NCE) regions, which often appear as T2/Fluid-Attenuated Inversion Recovery (FLAIR) hyperintensity or, in some cases, even as radiographically normal brain [11,12,13]. As a result, even gross total resection (GTR) inevitably leaves behind a substantial burden of infiltrative disease.

These infiltrating cell populations are thought to be the predominant source of tumor recurrence, as approximately 80–90% of glioblastoma recurrences occur within 2 cm of the original resection cavity [14,15,16]. This pattern suggests that removing only the CE tumor is insufficient for disease control and supports the extension of resection margins beyond the traditionally defined CE boundary. Supramaximal resection (SMR), a surgical approach that involves the deliberate removal of NCE or FLAIR-abnormal regions, has emerged as a promising strategy to reduce residual infiltrative tumor burden. Early retrospective and prospective studies suggest that SMR improves progression-free survival (PFS) and overall survival (OS) [17,18,19,20,21], yet the definitions and application of SMR vary widely across the literature.

The absence of consensus on how to define or measure SMR, combined with the emergence of novel imaging techniques, operative tools and functional mapping strategies, has led to significant heterogeneity in both clinical practice and patient outcomes. Furthermore, because infiltrative tumor cells exhibit distinct biological and molecular properties [22,23], surgical strategies must be integrated with novel molecular and immunologic therapies to effectively target residual infiltrative disease.

To address this complexity, we performed a systematic review of the literature evaluating supramaximal resection in glioblastoma. Given the variability in how SMR is defined and implemented, included studies were grouped and synthesized based on how resection beyond the CE boundary was characterized, including percentage-based FLAIR resection, volumetric residual tumor burden, metabolic or fluorescence-guided approaches, and anatomic or lobar resection strategies. With this approach, this systematic review aims to synthesize the biological and clinical rationale for SMR, evaluate the historical and contemporary evidence supporting its use, describe intraoperative techniques that enable safe and effective SMR, and outline emerging postoperative and systemic approaches to address infiltrative glioblastoma.

## 2. Methods

### 2.1. Literature Search Strategy

This systematic review was conducted in accordance with the PRISMA guidelines. A comprehensive literature search was performed using PubMed/MEDLINE, Embase, and Web of Science from database inception through March 2026. Search strategies were developed to identify studies evaluating SMR in glioblastoma. Keywords included combinations of: “glioblastoma”, “glioblastoma multiforme”, “GBM”, “supramaximal resection”, “supramarginal resection”, “supratotal resection”, “extent of resection”, “FLAIR”, “non-contrast-enhancing tumor”, “fluorescence-guided surgery”, “PET”, and “lobectomy”. The complete search string for each database is available in the Supplementary Materials File S1.

Search results from all databases were combined and duplicate records were removed using reference management software (Zotero v7.0.32). The final deduplicated dataset was used for screening.

### 2.2. Study Selection and Inclusion Criteria

**Figure 1:**
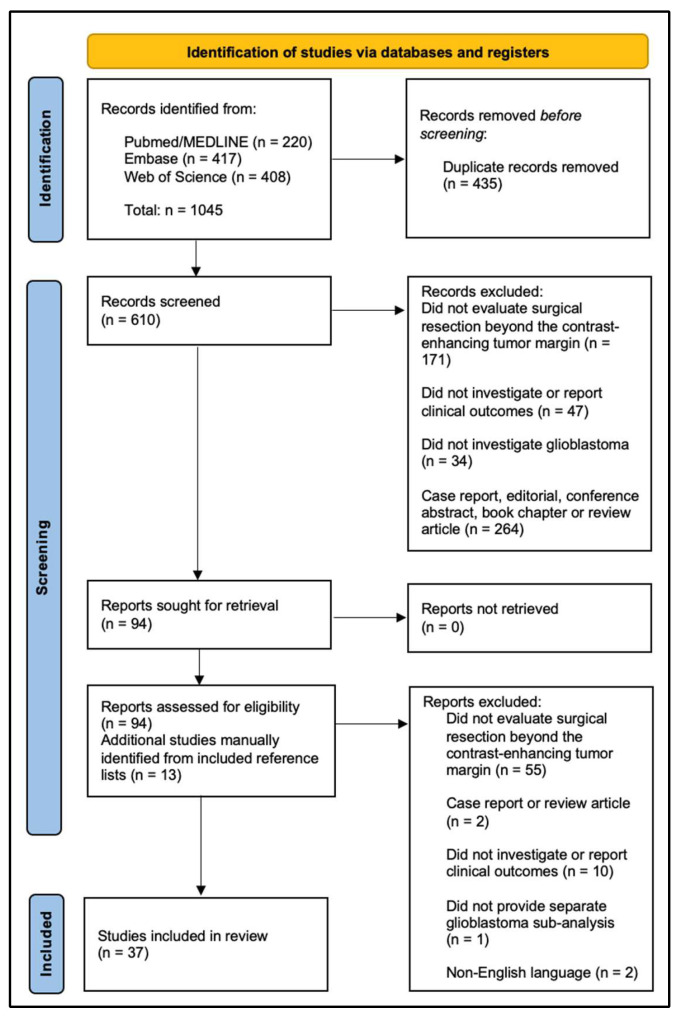
PRISMA flowchart detailing the systematic literature search.

The PRISMA flowchart is shown in Figure 1. A total of 1045 records were identified through database searches. After removal of duplicates, 610 records underwent title and abstract screening by two independent authors who were guided by predetermined inclusion criteria. Disagreement between the two screeners was resolved by discussion with a third author. Inclusion criteria included (1) investigated adult patients with glioblastoma, (2) evaluated surgical resection beyond the CE boundary, (3) included a quantitative or qualitative assessment of extent of resection, and (4) reported at least one clinical outcome such as progression-free survival, overall survival or recurrence pattern. Studies were excluded if they included non-glioblastoma tumors without a separate glioblastoma analysis, did not distinguish between CE and NCE tumor, did not evaluate resection beyond the CE boundary, or were case reports, review articles or conference abstracts.

There were 94 reports that progressed through screening and underwent full-text review. An additional 13 studies not identified in the systematic literature search were included after manual review of reference lists. Of the reports evaluated, 55 were excluded as they did not evaluate resection beyond the CE tumor margin, two were case reports or review articles, ten did not report clinical outcomes, one did not provide a separate sub-analysis for glioblastoma patients, and two did not have full-text available in English. This left a total of 37 studies that met our inclusion criteria and were included in this systematic review. Study bias was assessed qualitatively at the domain level and is included in Section 8.2.

### 2.3. Data Synthesis

Given the heterogeneity in definitions of SMR and study methodologies, a quantitative meta-analysis was not performed. Instead, studies were grouped and synthesized according to conceptual frameworks based on how resection beyond the contrast-enhancing margin was defined:

1. Percentage-based resection of FLAIR or NCE tumor;
2. Volume-based assessment of residual tumor burden;
3. Metabolic or fluorescence-guided resection;
4. Anatomic or lobar resection.

This framework was used to compare across studies and identify patterns in clinical outcomes in a structured fashion.

## 3. Biological and Clinical Rationale for Supramaximal Resection

### 3.1. Patterns of Tumor Infiltration Beyond Contrast Enhancement

A central challenge in glioblastoma surgery is that the radiographic boundary defined by CE on standard MRI does not capture the full extent of disease [11,12]. Although contrast enhancement reflects regions of blood–brain barrier disruption, microscopic tumor infiltration extends well into adjacent NCE tissue. These regions often correspond to T2/FLAIR hyperintensity, but numerous histologic and molecular studies have confirmed that even radiographically normal regions harbor infiltrating glioblastoma cells [13,24].

Glioblastoma spreads preferentially along established anatomic pathways. White matter tracts serve as the major route of invasion, enabling anisotropic spread along structures such as the corpus callosum, superior longitudinal fasciculus and corticospinal tract [25,26,27,28,29]. Perivascular spaces similarly facilitate long-range dissemination, allowing tumor cells to migrate along the vasculature [30,31]. Certain neuroanatomical regions, most notably the subventricular zone (SVZ), appear to be particularly susceptible to infiltrative growth and are consistently associated with more aggressive behavior and worse outcomes [32,33]. One proposed explanation is the unique stem-cell rich environment of the SVZ, which may enable glioblastoma cells to persist, proliferate or reconstitute tumor growth even after initial treatment. Conversely, regions such as the hippocampus are frequently spared from invasion [34], underscoring the possibility that local differences in cellular composition or microenvironment may either permit or restrict glioblastoma spread.

These patterns underscore a key limitation of surgical resection: even complete resection of the CE tumor inevitably leaves behind infiltrative disease in adjacent NCE tissue. As most glioblastomas recur within this infiltrative margin, the invasiveness of glioblastoma provides the foundational rationale for exploring SMR strategies.

### 3.2. FLAIR Abnormalities as Markers of Infiltrating Tumors

T2/FLAIR hyperintensity around the CE tumor represents a heterogeneous mixture of vasogenic edema, reactive changes, and infiltrating glioblastoma cells [35,36]. Although early radiographic interpretations viewed FLAIR abnormalities primarily as edema, mounting evidence has demonstrated that these regions frequently harbor substantial tumor burden, with cellular density gradually decreasing away from the CE rim and towards the periphery [12,13]. Several studies have shown that FLAIR signal intensity can correlate with tumor cell density [36,37], and that these infiltrating cells have unique properties that enable proliferative potential and treatment resistance [38,39].

Importantly, not all FLAIR signals carry the same oncologic significance. Areas of mass-like or nodular FLAIR abnormality tend to contain higher densities of infiltrating tumor cells and may have a greater prognostic impact than more diffuse or patchy changes [37,40]. These distinctions are clinically meaningful, suggesting that specific components of the FLAIR region, rather than the entire hyperintense zone, may be the most relevant targets for SMR [41]. Understanding the biological and radiographic heterogeneity of FLAIR abnormalities is therefore essential when evaluating which areas can be safely resected to offer the greatest possible benefit to each patient.

### 3.3. Infiltrative Biology of Glioblastoma

At the cellular and molecular level, the infiltrative margin of glioblastoma is biologically distinct from CE tumor. Rather than serving as a simple extension of the CE tumor, NCE regions exhibit a unique cellular and molecular organization shaped by their interaction with the surrounding brain parenchyma. NCE regions have been found to contain a higher proportion of non-neoplastic cells, including oligodendrocyte progenitors, astrocytes, and microglia, whereas the CE region is dominated by neoplastic tumor cells with less contribution from normal brain elements [12,42]. Crosstalk between infiltrating tumor and resident brain cells is thought to shape the transcriptional landscape and microenvironment of the NCE region [43,44], reinforcing its role as a biologically distinct tumor compartment.

These regional differences are accompanied by important transcriptional divergence. Infiltrative tumor cells beyond the CE margin exhibit increased expression of oligodendrocyte lineage markers and upregulation of neurodevelopmental pathways, including OLIG2 and ASCL1 [45]. In addition, infiltrative cells harbor distinct genomic and transcriptional alterations, including subclonal EGFR amplification and activation that are not uniformly represented within the CE tumor [11,46]. These NCE cells also demonstrate enhanced NOTCH signaling, elevated synaptic gene expression and activation of pathways involved in survival, migration and plasticity [22,47]. In contrast, CE regions demonstrate upregulation of genes involved with angiogenesis, extracellular matrix remodeling, and proliferation [45,48].

This spatially distinct diversion in gene expression translates into different functional behaviors. CE regions demonstrate higher proliferative activity, whereas infiltrative NCE tumor cells exhibit enhanced migratory capacity and increased resistance to cellular stress [49]. These complementary phenotypes are consistent with the “go-or-grow” paradigm, in which proliferative cells dominate the CE tumor while invasive, treatment-resistant cells migrate to the tumor margin [49,50]. Together, these features underscore the central role of the NCE region in glioblastoma progression and recurrence, and provide a biological rationale for further investigations into strategies designed to uniquely target these invasive, treatment-resistant NCE cells.

### 3.4. Hypothesized Impact of Infiltrative Margin Resection on Recurrence and Survival

The proposed utility of SMR is to reduce the residual burden of infiltrative glioblastoma cells that remain following surgical resection. Recurrence most commonly arises from the infiltrative margin surrounding the CE rim, and infiltrative NCE cells have additionally been shown to exhibit relative resistance to radiation and chemotherapy [51]. Thus, it is plausible that selectively extending resection into NCE or FLAIR-abnormal regions may delay local tumor recurrence and prolong disease control.

## 4. Historical and Early Evidence Supporting Supramaximal Resection

Although glioblastoma surgery was previously anchored to the goal of GTR of 100% of the CE tumor identified on preoperative MRI, several studies provided early clinical evidence that tumor burden beyond the CE margin has prognostic significance. These investigations, leveraging metabolic imaging, intraoperative fluorescence, and recurrence pattern analysis, collectively suggested that more extensive local resection could improve outcomes, even before the concept of SMR gained formal traction.

Metabolic and fluorescence-guidance approaches were some of the first modern techniques utilized to demonstrate that residual tumor outside the MRI-defined CE boundary adversely affects survival. Pirotte et al. showed that complete resection of metabolically active regions, as defined by PET tracer uptake, was associated with significantly longer survival in both glioblastoma and anaplastic glioma [52]. By comparison, complete resection of the CE region on MRI alone was not predictive of improved survival. Similarly, Aldave et al. demonstrated that glioblastoma patients with complete CE resection but residual intraoperative 5-aminolevulinic acid (5-ALA) fluorescence experienced significantly worse overall survival compared to patients where all fluorescent tissue was removed [53]. Together, these studies challenged the adequacy of CE-defined GTR and provided early biologic and clinical support for extending resection into NCE regions when safely feasible.

Additional indirect support came from analyses of recurrence patterns following extended resections. De Bonis et al. reported that resections extending 1–2 cm beyond the CE border were associated with lower rates of local recurrence and a higher frequency of distant recurrence, a pattern correlated with longer PFS and OS [54]. While not explicitly framed as SMR, these early studies suggested that removal of peritumoral tissue could alter glioblastoma growth and recurrence.

Building on this conceptual foundation, Li et al. provided one of the first large-scale, systematic evaluations of SMR in glioblastoma. In their single-institution cohort of 1229 patients, complete resection of CE tumor was associated with improved OS compared with subtotal resection [17]. Importantly, additional resection of ≥53.21% of the surrounding FLAIR abnormality conferred a further, statistically significant survival advantage without increased postoperative morbidity. This study explicitly reframed maximal safe resection to include targeted removal of NCE tumor, formally introducing SMR as a quantifiable and clinically impactful surgical strategy.

## 5. Modern Definitions and Contemporary Evidence

### 5.1. Framing the Contemporary Supramaximal Resection Literature

As interest in SMR has grown, so too has heterogeneity in how the concept is defined and applied across studies. Rather than representing a single operative strategy, SMR encompasses a spectrum of surgical strategies that extend resection beyond the CE tumor using different anatomic, radiographic, and volumetric criteria. Consequently, variability in reported outcomes across studies may reflect differences in definition, measurement, and patient selection rather than true inconsistency in the biological effect of extended resection.

Across the modern literature, SMR has been evaluated using several distinct frameworks. Some studies define SMR by the percentage of NCE or FLAIR abnormality resected, whereas others rely on absolute residual NCE tumor volume or binary thresholds distinguishing complete from incomplete resection. Still others describe anatomic or lobar extensions beyond radiographic abnormalities. These approaches are further shaped by methodological differences, including single-center versus multicenter design, retrospective versus prospective cohorts, and variable imaging segmentation techniques. All of these variables may contribute to the heterogeneity of reported results.

Importantly, many early SMR studies implicitly treated complete CE resection as the primary benchmark, with removal of NCE tumor considered an adjunct rather than a distinct surgical category. More recent work has shifted this paradigm by demonstrating that residual NCE tumor burden itself carries independent prognostic significance. When contemporary studies are grouped according to the tissue targeted beyond the CE margin and the metrics used to quantify resection, consistent survival patterns emerge. This framework provides a rational basis for synthesizing the SMR literature and sets the stage for examining margin-focused, volumetric, metabolic and anatomic approaches to SMR in this systematic review.

### 5.2. Percentage-Based Thresholds for FLAIR Abnormality Resection

Percentage-based thresholds for resection of NCE disease represent some of the earliest and most widely adopted quantitative frameworks for defining SMR. In these studies, SMR is typically defined as complete removal of the CE tumor followed by resection of a specified proportion of surrounding T2/FLAIR abnormality, with survival compared against predefined cutoff values. The seminal work by Li et al. established this paradigm by demonstrating that resection of ≥53.21% of peritumoral FLAIR abnormality after complete CE resection was associated with a significant prolongation of overall survival without an increase in postoperative neurological morbidity (Table 1) [17]. This study reframed maximal safe resection as a two-tiered construct, with CE tumor clearance as a prerequisite and FLAIR resection as an incremental survival modifier, providing the conceptual foundation for subsequent percentage-based threshold analyses.

Multiple single-institution studies subsequently proposed alternative, but similar, percentage cutoffs, reflecting differences in cohort composition, imaging methodology and surgical approach. Pessina et al. identified a significant OS threshold of 45% FLAIR resection [55], while Lu et al. reported improved PFS and OS with ≥25% FLAIR resection of tumor involving eloquent regions managed with awake surgical mapping [56]. Although the absolute thresholds varied, these studies converged on a consistent pattern: once complete CE resection was achieved, incremental removal of NCE disease to percentage-defined thresholds conferred additional survival benefit, with the exception of one single-institution series [57]. Importantly, none of these investigations reported an increase in permanent neurological deficits at their proposed thresholds, supporting the technical feasibility of moderate FLAIR extension in carefully selected patients.

As these investigations continued, larger and more molecularly refined cohorts reinformed the prognostic relevance of a percentage-based threshold for FLAIR resection, while simultaneously highlighting its limitations. In a cohort of 326 adults with newly diagnosed IDH-wildtype glioblastoma, Incekara et al. demonstrated that ≥30% resection of NCE tumor independently predicted improved OS after adjustment for age, MGMT promoter methylation, and adjuvant therapy [58]. Similarly, Vivas-Buitrago et al. identified approximately 20% FLAIR resection as the minimum threshold associated with survival benefit, with increasing benefit up to 50% resection and no further benefits beyond 60% [59]. Additional studies, including Otsuji et al. [60], Di et al. [61], Que et al. [62], and Polonara et al. [63], identified FLAIR resection thresholds producing survival benefit at ≥30%, ≥40%, ≥50%, and ≥90% respectively, while a recent study by Certo et al. identified that extent of FLAIR resection (EOFR) could independently predict PFS and OS, with each 1% increase in EOFR reducing mortality by 6.8% in IDH-wildtype glioblastoma patients [64,65]. These findings suggest that percentage-based thresholds capture a real biological signal but also underscore that no single cutoff universally defines the optimal extent of supramaximal resection.

Importantly, emerging data indicates that variability in reported thresholds may reflect underlying heterogeneity in tumor infiltration between study cohorts rather than inconsistency in surgical effect. Using a proliferation–diffusion mathematical model, Tripathi et al. demonstrated that the survival benefit associated with increasing FLAIR resection differed substantially according to tumor invasiveness, with diffuse and moderately diffuse tumors benefitting from greater resection percentages (30–90% and 10–50%, respectively) than nodular tumors (10–20%) [66]. This work provides a biological explanation for why threshold values across the literature have spanned from 20% to greater than 50%, and suggests that rigid percentage cutoffs may oversimplify a fundamentally continuous biological process. Taken together, the percentage-based threshold literature supports FLAIR resection as a meaningful extension of maximal safe surgery, while emphasizing that thresholds should be interpreted as heuristic guides rather than prescriptive targets.

**Table 1:** Clinical studies evaluating extended resection of FLAIR-abnormal tissue in glioblastoma. Summary of clinical studies identified the systematic literature review evaluating supramaximal resection strategies defined by percentage-based resection of non-contrast-enhancing T2/FLAIR abnormality beyond complete contrast-enhancing tumor removal in glioblastoma.

First Author and Year	PMID	Study Title	Patient Cohort	How SMR was Defined	SMR Threshold	Comparison Group	Key Outcomes	Effect Size
Li et al. 2016 [17]	26495941	The influence of maximum safe resection of glioblastoma on survival in 1229 patients: Can we do better than gross-total resection?	1229 adults with newly diagnosed or recurrent GBM undergoing ≥78% CE resection	Complete (100%) resection of CE tumor with additional resection of surrounding T2/FLAIR abnormality	≥53.21% FLAIR resection	<53.21% FLAIR resection	SMR: median OS 23.2 (95% CI 17.8–28.6) months. Comparison: median OS 18.7 (16.9–20.5) months	HR 1.53 (95% CI 1.33–1.77), *p* < 0.001
Pessina et al. 2017 [55]	28689368	Maximize surgical resection beyond contrast-enhancing boundaries in newly diagnosed glioblastoma multiforme: is it useful and safe? A single institution retrospective experience	282 adults with newly diagnosed GBM treated with surgery and chemoradiation	Complete resection of CE Tumor + 100% FLAIR abnormality removed	≥45% FLAIR resection	GTR with <45% FLAIR removal	SMR: median PFS 11.9 (95% CI 10.7–13.1) months and median OS 24.5 (18.9–30.1) months Comparison: median PFS 19.2 (12.9–25.4) months and median OS 15.7 (14.2–17.2) months	Median OS 24.5 vs. 16.2 months (absolute difference 8.3 months); *p* = 0.001. HR not reported.
Ahmadipour et al. 2019 [57]	30965373	Association of Surgical Resection, Disability, and Survival in Patients with Glioblastoma	565 adults with newly diagnosed GBM undergoing resection	Anatomical resection beyond CE tumor with removal of additional FLAIR abnormalities in non-eloquent regions	GTR + removal of additional FLAIR alterations	Gross total resection (≥95% reduction in CE tumor mass)	SMR was not associated with improved OS compared with GTR (median OS 15.0 vs. 17.1 months); on multivariate analysis, SMR was inferior to GTR	HR 1.27 (95% CI 0.74–2.17); *p* = 0.386
Incekara et al. 2020 [58]	32766140	The Association Between the Extent of Glioblastoma Resection and Survival in Light of MGMT Promoter Methylation in 326 Patients With Newly Diagnosed IDH-Wildtype Glioblastoma	326 adults with IDH-wildtype GBM	Volumetric assessment of CE and NCE tumor on postoperative MRI	≥30% resection of NCE (FLAIR) abnormality	<30% resection of NCE (FLAIR) abnormality	SMR: median OS 11.5 (95% CI 10.1–12.8) months. Comparison: median OS 8.9 (7.1–10.6) months	HR 0.71 (95% CI 0.53–0.93), *p* = 0.014
Lu et al. 2020 [56]	31899397	T2 Fluid-Attenuated Inversion Recovery Resection for Glioblastoma Involving Eloquent Brain Areas Facilitated Through Awake Craniotomy and Clinical Outcome	46 adults with newly diagnosed GBM in eloquent regions who underwent awake resection	After 100% CE resection, EOR of T2/FLAIR abnormality quantified volumetrically	≥25% FLAIR resection	<25% FLAIR resection	SMR: median PFS 15 (95% CI 11.7–18.3) months. and median OS 26 (21.5–30.5) months. Comparison: median PFS 6 (4.0–8.0) months. and median OS 12 (9.5–14.5) months.	Median OS 26 vs. 12 months. (absolute difference 14 months); *p* = 0.001. HR not reported.
Certo et al. 2021 [64]	33035343	FLAIRectomy in Supramarginal Resection of Glioblastoma Correlates with Clinical Outcome and Survival Analysis: A Prospective, Single Institution, Case Series.	68 adults with newly diagnosed GBM	100% CE tumor resection plus FLAIR-guided resection of NCE tumor	Extent of FLAIR resection analyzed as a continuous variable (no cutoff)	Outcomes compared to T1-based resection	SMR: mean PFS 17.4 months and mean OS 25.1 months; extent of FLAIR resection positively correlated with PFS and OS. T1-based resection showed weak/no correlation with survival.	FLAIR-based EOR correlated with OS (R^2^ = 0.68) and PFS (R^2^ = 0.46); *p* < 0.05. HR not reported.
Vivas-Buitrago et al. 2022 [59]	34087795	Influence of supramarginal resection on survival outcomes after gross-total resection of IDH-wild-type glioblastoma	101 adults with newly diagnosed IDH-wildtype GBM who underwent GTR and chemoradiation	Complete resection of CE tumor with calculated percentage resection of FLAIR abnormality beyond CE margin	≥20% FLAIR resection identified as minimum threshold with OS benefit. Observed up to 50% resection, no additional benefit beyond 60% resection	<20% FLAIR resection	SMR independently associated with improved OS (HR 0.99 per % increase, *p* = 0.02. ≥20% SMR: HR 0.56 (*p* = 0.01). ≥40% SMR: HR 0.52 (*p* < 0.01).	HR 0.56 (95% CI 0.35–0.89), *p* = 0.01
Tripathi et al. 2022 [66]	34715662	IDH-wild-type glioblastoma cell density and infiltration distribution influence on supramarginal resection and its impact on overall survival: a mathematical model	101 adults with newly diagnosed IDH-wildtype GBM who underwent GTR and chemoradiation	Volumetric percentage resection of T2/FLAIR hyperintensity beyond CE margin. Tumors stratified by proliferation/diffusion rate into nodular, moderately diffuse or highly diffuse.	Tumor invasiveness-dependent thresholds: Nodular: 10–20% Moderately Diffuse: 10–50% Highly Diffuse: 30–90% FLAIR resection	<% FLAIR threshold for each group	SMR independently associated with improved OS in moderately and highly diffuse tumors (HR 0.99 and 0.98 per % increase, *p* = 0.02 and = 0.04, respectively). OS benefit seen with SMR exceeding FLAIR thresholds for the three tumor classifications.	HR 0.99 (95% CI 0.98–0.99), *p* = 0.02 (moderately diffuse); HR 0.98 (95% CI 0.96–0.99), *p* = 0.04 (highly diffuse)
Di et al. 2023 [61]	35623362	Radical supramaximal resection for newly diagnosed left-sided eloquent glioblastoma: safety and improved survival over gross-total resection	102 adults with newly diagnosed left-sided eloquent GBM	Complete resection of CE tumor with additional resection of ≥40% preoperative FLAIR volume	GTR + resection of ≥40% preoperative FLAIR volume	GTR alone	SMR: median PFS 4.5 months and median OS 21.6 months Comparison: median PFS 3.6 months (*p* = 0.041) and median OS 15.5 months (*p* = 0.0098) Extent of FLAIR resection demonstrated dose-dependent improvement in PFS and OS	Median OS 21.6 vs. 15.5 months (absolute difference 6.1 months); *p* = 0.0098. HR not reported.
Polonara et al. 2023 [63]	36979717	Glioblastoma: A Retrospective Analysis of the Role of the Maximal Surgical Resection on Overall Survival and Progression Free Survival	64 adults with newly diagnosed GBM	100% CE tumor resection plus attempted complete resection of FLAIR abnormalities	Calculated threshold of ≥90% total resection (CE + FLAIR abnormality)	<90% total resection	SMR: median OS 18 months. Comparison: median OS 14 months (*p* = 0.018)	Median OS 18 vs. 14 months (absolute difference 4 months); *p* = 0.018. HR not reported.
Que et al. 2023 [62]	37783878	Supramaximal resection based on en-bloc technique reduces tumor burden and prolongs survival in primary supratentorial lobar glioblastoma	340 adults with supratentorial lobar GBM (including WHO IV astrocytoma, IDH-mutant and IDH-wt)	100% CE tumor resection plus > 50% resection of FLAIR abnormality	GTR + >50% FLAIR resection	GTR + <50% FLAIR resection or subtotal resection (<100% CE)	SMR: median PFS 22.5 (95% CI 18.0–27.1) months and median OS 27.6 (25.8–29.4) months Comparison: median PFS 14.3 (13.0–15.5) months and median OS 19.2 (17.4–20.9) months.	HR 0.41 (95% CI 0.31–0.55), *p* < 0.001
Otsuji et al. 2023 [60]	37423755	Supramaximal Resection Can Prolong the Survival of Patients with Cortical Glioblastoma: A Volumetric Study.	33 adults with newly diagnosed GBM with 100% CE tumor resection	Volumetric reduction in FLAIR abnormality beyond CE tumor after GTR	GTR with ≥ 30% FLAIR resection	GTR with <30% FLAIR resection	Median OS 69.9 months vs. 22.1 months (cortical tumors). Median OS 10.2 months vs. 27.9 months (deep-seated tumors)	Cortical tumors: Absolute difference 47.5 months (*p* = 0.0945).
Certo et al. 2025 [65]	40257266	Is FLAIRectomy Directly Correlated with Prolonged Survival in Glioblastoma? A Prospective National Multicenter Study on Correlation Between Extent of Tumor Resection and Clinical Outcome	150 adults with GBM or WHO grade IV astrocytoma; all deemed eligible for SMR	Extent of FLAIR resection (EOFR) beyond CE tumor; CE resection extent measured separately	Continuous EOFR model with no discrete cutoff	Higher vs. lower EOFR	EOFR independently predicted PFS and OS. Each 1% EOFR increase reduced mortality by 6.8% in IDH-wildtype GBM.	HR 0.93 (95% CI 0.92–0.95), *p* < 0.001

### 5.3. Volume-Based Approaches to Defining Supramaximal Resection

While percentage-based thresholds for FLAIR resection provided an important early quantitative framework, they are inherently constrained by dependence on preoperative tumor size and variability in imaging segmentation. Additional limitations, such as the imperfect distinction between infiltrative tumor and vasogenic edema, the effects of preoperative corticosteroid administration on FLAIR signal, and transient postoperative changes that may exacerbate edema, all further complicate interpretation of percentage-based metrics. These notable limitations motivated parallel investigation focused on absolute residual tumor volume as a more direct and biologically grounded measure of surgical success. Early studies adopting this approach conceptualized SMR not as a proportion of tissue removed, but as cytoreduction extending beyond the CE margin to minimize the absolute burden of residual infiltrative disease.

De Bonis et al. first demonstrated that resections extending 1–2 cm into surrounding normal-appearing white matter altered recurrence patterns, reducing local failure and increasing distant recurrence, a shift associated with prolonged survival in tumors located outside eloquent regions (Table 2) [54]. Similarly, Glenn et al. showed that removal of the CE tumor plus ≥1 cm of surrounding brain tissue in temporal lobe glioblastoma conferred marked improvements in both PFS and OS without increased complications rates [18]. Although neither study employed formal volumetric segmentation, both anticipated the core premise of volume-based SMR: the absolute quantity of residual infiltrative tumor, rather than the relative extent of resection alone, carries critical prognostic significance.

This conceptual shift was formalized through studies explicitly quantifying residual CE and NCE tumor volumes. In a landmark multicenter analysis, Molinaro et al. demonstrated that survival stratified strongly by residual tumor volume, even among patients achieving complete or near-complete CE resection [67]. Patients harboring minimal residual NCE disease experienced substantially prolonged survival across molecular subgroups. Moiraghi et al. extended this framework by using postoperative cavity volume relative to preoperative tumor volume as a categorical volumetric definition of supramaximal resection. Their study showed that resections yielding a postoperative cavity larger than the preoperative tumor volume were independently associated with improved OS [68]. Although most volumetric analyses converged on residual NCE burden as a key prognostic determinant, important outlier studies such as Mampre et al. highlight that volumetric definitions do not uniformly predict survival across all cohorts [69].

Building on this foundation, the Response Assessment in Neuro-Oncology (RANO) Resect group introduced a standardized, volume-based classification system for extent of resection in glioblastoma. In their pivotal 2023 report, Karschnia et al. proposed volumetric categories defined by absolute residual CE and NCE tumor volumes, most notably identifying ≤ 5 cm^3^ of residual NCE disease following complete CE resection as a key threshold associated with improved PFS and OS [70]. This framework reshaped SMR as a distinct surgical category defined by minimal residual infiltrative disease rather than arbitrary resection percentages. Subsequent validation studies have reinforced the robustness of this approach. Tropeano et al. confirmed that patients meeting RANO-Resect supramaximal criteria (Class 1: 100% CE resection plus ≤ 5 cm^3^ of residual NCE disease) experienced significantly prolonged survival, with outcomes inversely correlated with residual FLAIR volume [71].

Importantly, more recent analyses have demonstrated that the survival benefit associated with volume-based SMR is not uniform across all patient populations. Using RANO-Resect criteria in a large multicenter cohort, Teske et al. showed that SMR was associated with improved overall survival in younger patients, whereas this benefit was absent in patients aged ≥65 years, despite similar degrees of cytoreduction [72]. Similarly, Liu et al. reported that residual NCE tumor volume independently predicted PFS and OS, but that the magnitude of benefit varied according to age and clinical characteristics, suggesting diminishing returns from aggressive cytoreduction in older or more medically vulnerable patients [73]. These findings underscore that volume-based SMR, while biologically and prognostically meaningful [74], must be interpreted within a patient-specific framework that accounts for age, functional reserve, and competing risks.

**Table 2:** Studies using absolute residual tumor volume to define extent of supramaximal resection. Studies identified in the systematic review assessing the clinical value of supramaximal resection strategies defined by absolute residual tumor volume in patients with glioblastoma.

First Author and Year	PMID	Study Title	Patient Cohort	How SMR was Defined	SMR Threshold	Comparison Group	Key Outcomes	Effect Size
De Bonis et al. 2013 [54]	22537870	The influence of surgery on recurrence pattern of glioblastoma	131 adults with newly diagnosed supratentorial GBM located in non-eloquent areas	Resection extending beyond the tumor border into apparently normal white matter	1–2 cm margin of normal-appearing white matter beyond tumor border	Border resection with <0.5 cm margin at tumor border	SMR: median PFS 12 (95% CI 7–17) months and median OS 19 (16–22) months. Comparison: median PFS 9 (7–13) months and median OS 17 (12–24) months.	Median OS 19 vs. 17 months. (absolute difference 2 months); *p* = 0.15. HR not reported.
Glenn et al. 2018 [18]	29555603	An Examination of the Role of Supramaximal Resection of Temporal Lobe Glioblastoma Multiforme	32 adults with temporal lobe GBM without insular involvement undergoing initial resection	Complete (100%) resection of CE tumor plus resection of ≥1 cm of surrounding brain tissue beyond enhancement margin	≥1 cm margin of brain tissue resected beyond the CE border	<1 cm margin of brain tissue resected beyond the CE border	SMR: median PFS 15 (95% CI 9.9–20.1) months and median OS 24 (21.4–26.6) months Comparison: median PFS 7 (4.1–9.9) months and median OS 11 (0–22.7) months.	HR 0.17 (95% CI 0.05–0.57), *p* < 0.004
Mampre et al. 2018 [69]	30073866	Extending the resection beyond the contrast-enhancement for glioblastoma: feasibility, efficacy, and outcomes	245 adults with newly diagnosed GBM undergoing resection with standard chemoradiation	Surgical cavity volume on postoperative MRI > preoperative CE tumor volume	Postoperative cavity volume > preoperative CE tumor volume	Postoperative cavity volume ≤ preoperative CE tumor volume	Residual CE volume independently predicted recurrence and OS (HR 1.026 and 1.027, respectively, *p* ≤ 0.01). Postoperative FLAIR volume and % FLAIR resection were not associated with recurrence or survival	HR 1.027 (95% CI 1.007–1.032), *p* = 0.001
Molinaro et al. 2020 [67]	32027343	Association of Maximal Extent of Resection of Contrast-Enhanced and Non-Contrast-Enhanced Tumor with Survival Within Molecular Subgroups of Patients With Newly Diagnosed Glioblastoma	967 adults with newly diagnosed GBM. Development cohort: 761 Validation cohorts: 206	Volumetric resection of both CE and NCE tumor, quantified as percent resection and residual NCE volume	Complete or near-complete (>77%) resection of CE + ≤5.4 mL residual NCE tumor	Complete or near-complete (>77%) resection of CE + >5.4 mL residual NCE tumor	SMR: median OS 31.7 (95% CI 22.2–56.2) months. Comparison: median OS 17.9 (16.4–19.7) months.	Median OS 31.7 vs. 17.9 months. (absolute difference 13.8 months.); *p* < 0.001. HR not reported.
Moiraghi et al. 2021 [68]	34200799	Feasibility, Safety and Impact on Overall Survival of Awake Resection for Newly Diagnosed Supratentorial IDH-wildtype Glioblastomas in Adults	453 adults with newly diagnosed supratentorial IDH-wildtype GBM	Complete CE resection plus postoperative cavity volume exceeding preoperative tumor volume.	Categorial volumetric definition: postoperative cavity volume greater than preoperative tumor volume	Postoperative cavity volume not exceeding preoperative tumor volume	SMR: median OS 36.0 (95% CI 23.0-not reached) months. Comparison: median OS 22.0 (19.4–24.9) months. Awake surgery increased likelihood of STR and independently improved OS	HR 0.27 (95% CI 0.12–0.62), *p* = 0.0021
Karschnia et al. 2023 [70]	35961053	Prognostic validation of a new classification system for extent of resection in glioblastoma: A report of the RANO resect group	1008 adults with newly diagnosed IDH-wildtype GBM	Defined volumetrically based on residual NCE tumor after resection. Introduced RANO categories for extent of resection in glioblastoma	100% CE resection + ≤5 cm^3^ residual non-CE tumor ± ≥60% reduction in NCE volume	100% CE resection + <60% NCE ± >5 cm^3^ NCE residual OR ≥95% CE resection ± ≤1 cm^3^ CE	SMR: median PFS 11 (95% CI 9–13) months and median OS 29 (20–44) months Comparison: median PFS 9 (8–10) months and median OS 19 (17–20) months.	HR 1.58 (95% CI 1.1–2.3), *p* = 0.013
Tropeano et al. 2024 [72]	38676720	Supramaximal resection: retrospective study on IDH-wildtype Glioblastomas based on the new RANO-Resect classification	117 adults with newly diagnosed IDH-wildtype GBM treated with resection and chemoradiation	Uses 2022 RANO-Resect criteria based on absolute residual tumor volume	100% CE resection + ≤5 cm^3^ residual non-CE tumor	100% CE resection + >5 cm^3^ NCE residual	SMR: median PFS 20 (Q1-Q3 12–26) months and median OS 24 (17–37) months Comparison: median PFS 13 (8–16) months and median OS 17 (11–26) months Survival inversely correlated with residual FLAIR volume (r = −0.28)	HR 0.207 (95% CI 0.098–0.436); *p*-value not reported.
Teske et al. 2025 [72]	41137668	Associations of supramaximal resection with outcome in glioblastoma across 3 age groups: a report of the RANO resect group	1260 adults with newly diagnosed IDH-wildtype GBM. 512 >65 years, 748 <65 years.	Volumetric RANO-Resect classification based on residual tumor volume	100% CE resection + ≤5 cm^3^ residual non-CE tumor	100% CE resection + >5 cm^3^ NCE residual	SMR associated with improved OS only in patients <65 years (40 months vs. 20 months.). No OS benefit over maximal CE resection in those >65 years.	HR 0.50 (95% CI 0.38–0.65), *p* = 0.001 (≤65 years only)
Zigiotto et al. 2025 [75]	40539789	Maximizing Tumor Resection and Managing Cognitive Attentional Outcomes: Measures of Impact of Awake Surgery in Glioma Treatment	64 adults with gliomas undergoing complete CE resection with awake or asleep surgery. 34 IDH-wildtype GBM	Volumetric analysis of residual NCE volume beyond CE margin	Residual NCE volume <40 cc	Residual NCE volume >40 cc	Awake surgery achieved greater NCE resection (68.9% vs. 42.7%) and lower NCE residual volume (13.4 vs. 43.1 cc). In IDH-wildtype GBM, awake surgery was associated with longer OS (mean 29.6 vs. 18.5 months)	Median OS 29.6 vs. 18.5 months (absolute difference 11.1 months); *p* < 0.05. HR not reported.
Liu et al. 2025 [73]	39842498	Aggressive resection of non-contrast-enhanced tumor provides varying benefits to glioblastoma, IDH-wildtype patients based on different clinical characteristics	155 adults with newly diagnosed IDH-wildtype GBM with complete CE resection. Additional prospective validation cohort of 128.	Volumetric residual NCE tumor measured as defined by FLAIR hyperintensity beyond CE margin, based on RANO-Resect categories	Residual NCE volume thresholds ≤43.27 mL and ≤5.27 mL	Residual NCE volumes not meeting described thresholds	Residual NCE volume independently predicted OS and PFS (per mL HR 1.016, *p* = 0.002). RANO Class 1 had median PFS and OS of 23.3 and 32.7 months compared to 13.8 and 21.2 months for Class 2A	Median OS 32.7 vs. 21.2 months (absolute difference 11.5 months); *p* = 0.022. HR not reported.
Tang et al. 2025 [74]	4074606	Impact of peri-tumoral resection on survival in primary glioblastoma	126 adults with newly diagnosed IDH-wildtype GBM in non-eloquent regions	Volumetric peri-tumoral resection (PTR) defined as resection cavity volume beyond CE tumor	PTR > 1.74 cm^3^ beyond CE tumor	PTR < 1.74 cm^3^	SMR: mean OS 21.6 months and mean PFS 12.9 months. Comparison: mean OS 16.8 months and mean PFS 8.9 months.	Mean OS 21.6 vs. 16.8 months; *p* < 0.01. HR 0.27 (95% CI 0.13–0.56)

### 5.4. PET- and Fluorescence-Guided Supramaximal Resection

While MRI-based approaches define supramaximal resection using structural surrogates of tumor infiltration, metabolic imaging modalities offer a complementary strategy by identifying biologically active tumor that extends beyond conventional anatomic boundaries. Positron emission tomography (PET) using amino acid tracers and intraoperative 5-ALA fluorescence both exploit metabolic differences between tumor and normal brain, frequently revealing regions of viable tumor that are not visible on contrast-enhanced MRI. As such, metabolic imaging-guided SMR reframes the surgical target as metabolically active disease rather than radiographic abnormality alone, providing a biologically informed rationale for extending resection margins.

Early evidence supporting this paradigm emerged from PET-guided surgical series. Pirotte et al. demonstrated that complete resection of metabolically active tumor, defined by ^18^F-Fluorodeoxyglucose (FDG) or ^11^C-methionine PET uptake, was strongly associated with improved survival in high-grade gliomas, whereas complete resection of MRI CE alone did not confer a similar benefit (Table 3) [52]. Subsequent studies by Pessina et al. and Hirono et al. using ^11^C-methionine PET reinforced these findings, showing that residual PET-avid tumor following apparent GTR independently predicted worse outcomes [76,77]. Importantly, these studies established that metabolic tumor burden represents a distinct and prognostically meaningful compartment that is not reliably captured by standard MRI, supporting the concept of PET-defined SMR.

Parallel advances in intraoperative fluorescence-guided surgery further expanded the role of metabolic targeting in SMR. Aldave et al. demonstrated that residual 5-ALA fluorescence following complete MRI-defined CE resection was associated with significantly shorter OS, indicating that fluorescent tumor beyond the CE margin harbors clinically relevant disease [53]. Eyüpoglu et al. extended this concept through a dual intraoperative visualization approach integrating 5-ALA fluorescence with intraoperative MRI, achieving “supra-complete” resections associated with prolonged survival without increased neurological morbidity [78].

More recent studies combining metabolic modalities have further strengthened this framework. Müther et al. demonstrated that resection of 5-ALA signal beyond gadolinium enhancement resulted in lower residual FET-PET volumes and improved survival, even when postoperative MRI suggested complete resection [79]. Similarly, Della Pepa et al. evaluated the integration of 5-ALA fluorescence and contrast-enhanced ultrasound (CEUS) and found that combined multimodal guidance was associated with the highest median EOR and the greatest rates of SMR compared with approaches using a single modality alone [80].

Collectively, these studies establish metabolic imaging-guided SMR as a biologically grounded extension of maximal safe resection, capable of identifying and targeting infiltrative tumor compartments that escape conventional MRI-based definitions. By prioritizing viable tumor over anatomic appearance alone, metabolic approaches provide a mechanistic bridge between structural imaging, volumetric cytoreduction, and oncologic outcome. However, their application remains constrained by availability, tracer specificity, and integration into routine surgical workflows, underscoring the need for continued refinement and standardization as these techniques move toward broader clinical adoption.

**Table 3:** Investigations of 5-ALA and PET-guided surgical resection beyond MRI-defined tumor margins. Clinical studies identified in systematic literature review evaluating supramaximal resection strategies guided by metabolic imaging modalities, including PET and intraoperative 5-ALA-based fluorescence, to target tumor beyond CE MRI border in glioblastoma.

First Author and Year	PMID	Study Title	Patient Cohort	How SMR was Defined	SMR Threshold	Comparison Group	Key Outcomes	Effect Size
Pirotte et al. 2009 [52]	19240609	Positron emission tomography-guided volumetric resection of supratentorial high-grade gliomas: a survival analysis in 66 consecutive patients	66 adults with supratentorial HGG (31 glioblastoma) undergoing initial resection	Surgical target volume defined by PET tracer uptake; goal was complete resection of abnormal metabolic signal	Complete PET tracer uptake resection	Residual PET uptake present on post-operative scan	Complete PET resection significantly improved survival in glioblastoma (HR 0.532, *p* < 0.0001) Complete MRI CE resection did not correlate with survival (*p* = 0.6806)	HR 0.532, *p* < 0.0001
Aldave et al. 2013 [53]	23685503	Prognostic Value of Residual Fluorescent Tissue in Glioblastoma Patients After Gross Total Resection in 5-Aminolevulinic Acid-Guided Surgery	52 adults with newly diagnosed GM with complete CE resection and intraoperative 5-ALA	Intraoperative assessment of residual visible 5-ALA fluorescence	Residual fluorescence absent	Residual fluorescence present	SMR: median OS 27.0 (95% CI 22.4–31.6) months. Comparison: median OS 17.5 (12.5–22.5) months. Residual fluorescence independently predicted worse survival on multivariate analysis (HR 2.5, *p* = 0.041)	HR 2.5, *p* = 0.041
Eyüpoglu et al. 2016 [78]	27036027	Supra-complete surgery via dual intraoperative visualization approach (DiVA) prolongs patient survival in glioblastoma	105 adults with glioblastoma undergoing complete CE resection	Complete resection of CE tumor using both intraoperative MRI with functional neuronavigation and resection of all 5-ALA fluorescence signal	Complete elimination of all 5-ALA signal, corresponding to resection beyond CE margin	GTR alone	SMR: median OS 18.5 (3–44) months. Comparison: median OS 14 (3–24) months.	HR 0.449 (95% CI 0.289–0.696), *p* = 0.0004
Müther et al. 2019 [79]	31215632	5-Aminolevulinic Acid Fluorescence-Guided Resection of 18F-FET-PET Positive Tumor Beyond Gadolinium Enhancing Tumor Improves Survival in Glioblastoma	31 adults with newly diagnosed GBM undergoing 5-ALA guided resection followed by chemoradiation	^18^F-FET-PET volume on postoperative PET scan	Residual FET-PET volume ≤ 4.3 cm^3^	>4.3 cm^3^ residual FET-PET volume	Patients with ≤ 4.3 cm^3^ residual FET-PET had significantly longer PFS and OS (HR 1.02 and 1.04 per cm^3^, respectively). Complete resection of 5-ALA fluorescence resulted in lower postoperative FET-PET volumes and improved survival, even when MRI showed complete resection.	HR 1.03 (95% CI 1.01–1.059), *p* = 0.006
Della Pepa et al. 2020 [80]	32186345	5-Aminolevulinic Acid and Contrast-Enhanced Ultrasound: The Combination of the Two Techniques to Optimize the Extent of Resection in Glioblastoma Surgery	230 adults with newly diagnosed GBM undergoing initial resection with intraoperative 5-ALA and contrast-enhanced ultrasound (CEUS)	Surgical cavity volume on postoperative MRI > preoperative CE tumor volume	Postoperative cavity volume > preoperative CE tumor volume	Four intraoperative strategies: 5-ALA + CEUS, 5-ALA alone, CEUS alone, conventional surgery only	Combined 5-ALA + CEUS achieved highest median EOR and highest rate of SMR (69.8%). EOR independently predicted PFS and OS. 5-ALA groups associated with improved OS compared to conventional surgery	HR 0.518 (95% CI 0.305–0.881), *p* = 0.015
Pessina et al. 2021 [76]	34070698	Role of 11C Methionine Positron Emission Tomography (11CMETPET) for Surgery and Radiation Therapy Planning in Newly Diagnosed Glioblastoma Patients Enrolled into a Phase II Clinical Study	93 adults with newly diagnosed, IDH-wildtype GBM enrolled in prospective Phase II trial who underwent surgical resection with pre- and postoperative MRI and PET available	Postoperative metabolic tumor burden; extent of residual tumor beyond CE border using 11C-Methionine PET	Absence of residual metabolic tumor volume on postoperative 11C-Methionine PET	Presence of residual metabolic tumor volume on postoperative 11C-Methionine PET	SMR: median OS 25 (20–34) months. Comparison: median OS 14 (12–18) months.	HR 0.4800 (95% CI 0.3772–0.6109), *p* < 0.0001
Hirono et al. 2021 [77]	34267303	Oncological and functional outcomes of supratotal resection of IDH1 wild-type glioblastoma based on 11C-methionine PET: a retrospective, single-center study	30 adults with newly diagnosed IDH1-wildtype GBM with GTR of CE tumor and underwent preoperative 11C-methionine PET	Complete resection of CE tumor and all regions of increased methionine uptake on 11C-Met PET	GTR + resection of entire PET signal	GTR with residual PET signal present	SMR: Median OS not yet reached (95% CI 30.5-not estimable) months. Comparison: median OS 18.5 (14.2–35.1) months. SMR associated with reduced local recurrence compared to GTR (0% vs. 80%)	Median OS not reached vs. 18.5 months (absolute difference not estimable); *p* = 0.03. HR not reported.

### 5.5. Anatomic and Lobar Extensions of Supramaximal Resection

Beyond radiographic and volumetric definitions, SMR has also been conceptualized through anatomic extension of resection to encompass entire lobar units harboring infiltrative disease. In this framework, SMR is achieved not by targeting a predefined percentage or volume of NCE tissue, but by performing an anatomic lobectomy following complete resection of the CE tumor. This strategy is grounded in the recognition that glioblastoma infiltration frequently respects white-matter and lobar boundaries, and that removal of functionally expendable cortex within non-eloquent frontal, temporal or occipital regions may allow for more comprehensive clearance of infiltrative tumor while maintaining neurological function.

Early single-institution studies provided compelling evidence supporting this approach in carefully selected patients. Roh et al. demonstrated that patients with IDH-wildtype glioblastoma confined to non-dominant frontal or temporal lobes who underwent lobectomy following complete CE resection experienced marked improvements in both PFS and OS compared with patients undergoing GTR alone, without significant differences in postoperative KPS scores (Table 4) [19]. Similarly, Shah et al., using a propensity-matched analysis, reported significantly prolonged survival in patients treated with lobectomy compared with GTR for non-eloquent glioblastoma, again without increased perioperative morbidity [20]. These findings were reinforced by Yoo et al., who showed that supratotal resections incorporating lobectomy not only improved survival but also altered recurrence patterns, with reduced local recurrence and increased distant failure, an observation consistent with more effective local disease control [81]. At a broader level, Zheng et al. synthesized available institutional series in a systematic review and individual participant meta-analysis, demonstrating that lobectomy was associated with superior OS and PFS compared with GTR, without worsening functional outcomes or complication rates [82]. Together, these studies support anatomic SMR as a robust extension of maximal safe resection in anatomically favorable tumors located in non-eloquent regions.

However, the generalizability of lobectomy-based SMR is tempered by population-level data highlighting important limitations. In a large SEER-based analysis of geriatric glioblastoma patients, Lopez-Rivera et al. found that while GTR and SMR were associated with improved survival compared with biopsy or subtotal resection, SMR (defined by surgical codes for lobectomy) did not confer a clear survival advantage over GTR alone in patients aged ≥65 years [83]. Furthermore, Borger et al. demonstrated that anterior temporal lobectomy (ATL) was capable of superior postoperative seizure freedom compared to GTR alone in temporal lobe glioblastoma, although without an accompanying survival benefit [84]. These findings underscore the influence of age, functional reserve, and selection bias in interpreting outcomes associated with aggressive anatomic resections. Collectively, the anatomic SMR literature suggests that lobectomy can offer substantial oncologic benefit in highly selected patients with non-eloquent, lobar disease, but also reinforces that its application must remain individualized, balancing anatomical opportunity against patient-specific risk.

**Table 4:** Clinical studies of lobar-based surgical extension beyond gross total resection. Summary of articles identified in the systematic review assessing outcomes following lobar or compartment-based surgical extension beyond gross total resection in glioblastoma.

First Author and Year	PMID	Study Title	Patient Cohort	How SMR was Defined	SMR Threshold	Comparison Group	Key Outcomes	Effect Size
Roh et al. 2020 [19]	30835701	Survival benefit of lobectomy over gross-total resection without lobectomy in cases of glioblastoma in the non-eloquent area: a retrospective study	40 adults with newly diagnosed IDH-wt GBM confined to nondominant frontal or temporal lobe undergoing complete resection	Complete resection of CE tumor followed by anatomic lobectomy	GTR + Lobectomy	GTR alone	SMR: median PFS 30.7 (95% CI 4.3–57.1) months and median OS 44.1 (25.1–63.1) months. Comparison: median PFS 11.5 (8.8–14.2) months and median OS 18.7 (14.3–23.1) months.	HR 0.247 (95% CI 0.086–0.704), *p* = 0.009
Shah et al. 2020 [20]	32627128	Survival benefit of lobectomy for glioblastoma: moving towards radical supramaximal resection	69 adults with newly diagnosed non-eloquent GBM (right frontal, temporal or occipital, left occipital) undergoing initial resection	Complete resection of CE tumor followed by anatomic lobectomy	GTR + Lobectomy	GTR alone	SMR: median PFS 17.2 (95% CI 11.5–22.9) months and median OS 30.7 (20.5–40.9) months. Comparison: median PFS 8.1 (6.3–9.9) months and median OS 14.1 (9.4–18.8) months.	Median OS 30.7 vs. 14.1 months (absolute difference 16.6 months); *p* = 0.019. HR not reported.
Lopez-Rivera et al. 2021 [83]	33454497	Extent of resection and survival outcomes of geriatric patients with glioblastoma: Is there benefit from aggressive surgery?	17,820 glioblastoma patients with age ≥65 years in SEER database	Surgical coding using SEER database	Administrative via registry codes (primarily lobectomy); no quantitative threshold	GTR alone via registry codes	Both SMR (HR 0.65) and GTR (HR 0.61) associated with improved OS vs. biopsy/STR. No OS advantage of SMR over GTR	SMR vs. biopsy/local excision: HR 0.65 (95% CI 0.62–0.68), *p* < 0.0001 GTR vs. biopsy/local excision: HR 0.61 (95% CI 0.58–0.65), *p* < 0.0001
Borger et al. 2021 [84]	33554293	Seizure outcomes in temporal glioblastoma surgery: lobectomy as a supratotal resection regime outclasses conventional gross-total resection	33 adults with temporal lobe GBM and preoperative seizures undergoing GTR or anterior temporal lobectomy (ATL)	Complete CE tumor resection followed by ATL	GTR + Lobectomy	GTR alone	SMR: postoperative seizure freedom 100%, median OS 22 months. Comparison: postoperative seizure freedom 50%, median OS 26 months. (*p* = 0.34)	Seizure freedom 100% vs. 50% (*p* = 0.002). HR not reported.
Yoo et al. 2022 [81]	34972087	Patterns of recurrence according to the extent of resection in patients with IDH-wild-type glioblastoma: a retrospective study	358 adults with newly diagnosed IDH-wildtype GBM	Complete resection of CE tumor plus additional lobectomy in non-eloquent regions	GTR + Lobectomy	GTR alone	SMR: median PFS 35.5 months and median OS 44.7 months. Comparison: median PFS 14.3 months and median OS 21.6 months.	Median OS 44.7 vs. 21.6 months (absolute difference 23.1 months); *p* < 0.0001. HR not reported.
Zheng et al. 2023 [82]	37487449	Lobectomy versus gross total resection for glioblastoma multiforme: A systematic review and individual-participant data meta-analysis	Systematic review; 286 adult glioblastoma patients (59% GTR, 41% lobectomy)	Complete resection of CE tumor followed by anatomic lobectomy	GTR + Lobectomy	GTR alone	SMR: median PFS 15.4 (95% CI 10.7–18.8) months and median OS 25.1 (20.4–30.7) months. Comparison: median PFS 7.3 (6.2–8.0) months and median OS 12.1 (10.2–15.8) months. No differences in KPS or complication rates.	Median OS 25.1 vs. 12.1 months (absolute difference 13.0 months); *p* < 0.001. HR not reported.

## 6. Intraoperative Strategies to Achieve Supramaximal Resection

### 6.1. Preoperative Planning and Imaging-Based Risk Stratification

Advanced MRI techniques play a central role in enabling safe and effective SMR by defining both the opportunity and the functional constraints of extended surgery before a patient ever enters the operating room. Preoperative volumetric analysis of CE and NCE tumor components allows surgeons to quantify baseline tumor burden, establish cytoreductive targets, and anticipate the potential magnitude of residual disease. Because EOR demonstrates a non-linear relationship with survival [67,70], precise volume characterization is becoming increasingly important, not only for postoperative outcome assessment, but also for preoperative risk–benefit modeling when considering SMR. In this context, advanced imaging reframes SMR as a planned surgical strategy rather than an intraoperative decision.

Functional imaging further refines this planning process by delineating eloquent cortex and critical white matter pathways that define the true limits of safe resection. Diffusion tensor imaging (DTI) with tractography enables visualization of major white matter bundles underlying or adjacent to infiltrative tumor, while functional MRI (fMRI) localizes motor, sensory, and language networks that may constrain extended resection [85]. Recent work by Nichols et al. utilized whole-brain tractography to show that preoperative resectability metrics derived from tumor-tract overlap can accurately predict EOR, residual tumor burden, and OS using preoperative imaging alone [86]. Additionally, a systematic review and meta-analysis by Luna et al. demonstrated that preoperative fMRI mapping was associated with a 75% reduction in the odds of persistent postoperative neurological deficits (OR 0.25, *p* < 0.001) and higher KPS compared to surgery without fMRI [87]. Together, these modalities inform decisions regarding surgical trajectories, the applicability of awake craniotomy, and the feasibility of SMR for each patient individually. Although subject to limitations from edema, infiltration, and brain shift, preoperative functional imaging provides important context for tailoring resection goals based on tumor location, patient functional reserve, and neurological risk. By identifying where SMR is feasible, unsafe, or unlikely to confer benefit, advanced imaging supports individualized surgical planning that prioritizes neurological preservation while maximizing oncologic impact.

### 6.2. Neuronavigation, Brain Shift and Intraoperative Imaging

Conventional neuronavigation based on preoperative imaging datasets including fMRI and DTI remains a foundational tool for intraoperative orientation and lesion localization during glioblastoma resection. In a recent multicenter cohort study of supratentorial gliomas, neuronavigation was associated with a 57% relative increase in GTR rates (56% vs. 35.5%) and a 79% reduction in postoperative neurological deficits compared with conventional microsurgery [88], underscoring its safety and utility. However, the accuracy of neuronavigation becomes increasingly unreliable after dural opening, cerebrospinal fluid loss, and tissue resection: a phenomenon known as “brain shift”.

Imaging studies have shown that brain shift is not a static event but rather a continuous dynamic process that evolves throughout surgery, with estimates suggesting average cortical surface displacement of 4.6 mm after dural opening and shifts at the deep tumor margin averaging 5.1 mm [89]. Furthermore, Nimsky et al. demonstrated that these shifts can reach maximum displacements of up to 24 mm for cortical structures, and that 66% of all cases in their series exceeded 3 mm of shift at the deep tumor margin [90]. Consistent with these findings, a critical review of intraoperative guidance strategies demonstrated that neuronavigation alone achieves GTR in only 31–36% of glioma cases. However, with the addition of real-time intraoperative imaging modalities such as fluorescence guidance, ultrasound or intraoperative MRI (iMRI), GTR rates increased to approximately 70–85% [91].

Further studies have similarly supported the utility of intraoperative imaging to complement conventional neuronavigation. In a prospective randomized controlled trial, Senft et al. demonstrated that iMRI guidance increased rates of complete resection from 68% to 96%, with additional tumor resection performed in one-third of cases due to iMRI findings and without an increase in postoperative neurological deficits [92]. Additional studies have similarly validated the important role of iMRI in increasing EOR [93,94,95]. Although iMRI is estimated to prolong surgery by 42 min on average [96], recent evidence suggests that iMRI may even provide more accurate assessment than 48 h postoperative MRI due to reduced surgically induced contrast enhancement that can mimic residual tumor [97]. Intraoperative ultrasound (IOUS) in combination with 5-ALA guidance is another surgical tool that can be utilized for identifying residual disease, with a recent report by Aibar-Durán et al. demonstrating 91% sensitivity and 86% specificity [98].

Together, these data demonstrate that static neuronavigation alone is insufficient for maintaining spatial accuracy during glioblastoma resection, but that integration of real-time intraoperative imaging can help to compensate for brain shift and increase EOR without affecting neurological morbidity.

### 6.3. Fluorescence-Guided and Optical Techniques

As detailed in Section 5.4 and summarized in Table 3, both PET and 5-ALA fluorescence have been extensively studied as metabolic adjuncts to SMR, demonstrating that biologically active tumor extends beyond CE margins. Building on this foundation, intraoperative fluorescence has been increasingly leveraged as a tool for intraoperative margin interrogation. Biochemically, 5-ALA serves as a precursor in the heme biosynthesis pathway and preferentially accumulates as fluorescent protoporphyrin IX within glioma cells due to altered enzymatic activity and reduced ferrochelatase-mediated conversion to heme. At the tumor–brain interface, Della Puppa et al. demonstrated that strong 5-ALA fluorescence at the margin was highly predictive of residual tumor and outperformed fluorescein in identifying infiltrative disease (PPV 94% vs. 87%) [99]. Similarly, Yano et al. observed 5-ALA-positive but fluorescein-negative regions in seven of eight patients, supporting the ability of 5-ALA to detect metabolically active tumor beyond areas of blood–brain barrier disruption [100]. In a case series of 100 patients, Pesaresi et al. reported that combined 5-ALA and fluorescein guidance achieved GTR in 96% of cases and SMR in 11% [21], highlighting the potential complementary value of dual-fluorescence approaches.

Despite these advantages, important limitations constrain the interpretability of 5-ALA fluorescence at the infiltrative margin. Multiple studies have demonstrated that fluorescence is neither perfectly specific nor sensitive for tumor. Ricciardi et al. showed that false-positive fluorescence is more common in recurrent glioblastoma, frequently reflecting inflammation or treatment-related changes rather than viable tumor [101]. Lau et al. reported that absence of fluorescence carries a poor negative predictive value, with tumor absent from non-fluorescent biopsies in only 35.4% of samples [102]. Histopathological correlation studies by Kiesel et al. further underscored this limitation, demonstrating that infiltrative tumor was present in 49% of non-fluorescent specimens [103]. Collectively, these findings emphasize that while 5-ALA is a powerful intraoperative tool for visualizing metabolically active tumor, fluorescence alone cannot reliably delineate the true infiltrative boundary, motivating the development of complementary optical and quantitative techniques.

One novel imaging approach, pH-weighted amine chemical exchange saturation transfer echo planar imaging (CEST-EPI), has been shown to visualize regions of infiltrative disease and predict tumor recurrence. This technique detects extracellular acidification generated by metabolically active tumor, with elevated signal correlating with tumor cell density, proliferative activity and PFS [104,105]. The safety and efficacy of supramaximal resection guided by CEST-EPI signal is now an ongoing Phase I clinical trial (NCT06176066). Other approaches such as time-resolved fluorescence spectroscopy enable real-time discrimination of tumor, edema, and normal brain based on fluorescent decay signatures, with early studies demonstrating 83% sensitivity and 93% specificity for high-grade gliomas [106]. Confocal laser endomicroscopy and optical coherence tomography represent additional emerging modalities that provide cellular-resolution “optical biopsy” at the infiltrative margin [107]. Near-infrared fluorescence imaging has also been associated with 18–22% higher rates of GTR in early clinical series [108], while interstitial photodynamic therapy using stereotactically implanted diffuser fibers extends optical techniques beyond visualization toward localized cytotoxic treatment [109]. Together, these technologies reflect a shift toward multimodal intraoperative guidance that may complement fluorescence-guided SMR and refine margin assessment in glioblastoma.

### 6.4. Functional Mapping, Electrophysiologic Monitoring, and Awake Surgery

Additional intraoperative strategies enable SMR to be performed within patient-specific boundaries, allowing aggressive cytoreduction while preserving critical cortical and subcortical networks. Intraoperative stimulation mapping (ISM) using direct electrical stimulation (DES) of the cortex and subcortical white matter remains the gold standard for real-time functional localization, defining resection stop-points based on immediate physiological responses rather than anatomic landmarks alone [110]. Comparative studies of preoperative and intraoperative mapping highlight the limitations of preoperative imaging alone: Spena et al. demonstrated strong concordance between fMRI and ISM for sensorimotor cortex (92.3%) but poor correlation for language areas (42.8%), while DTI systematically underestimated the presence of functional fibers within and adjacent to tumor [111]. These findings underscore that while preoperative modalities are valuable for surgical planning, as described above, they cannot reliably define resection limits without intraoperative confirmation.

Accordingly, integration of preoperative mapping with ISM and continuous monitoring has emerged as the optimal strategy for balancing EOR and functional preservation, particularly for tumors involving eloquent structures. Baig Mirza et al. showed that combined preoperative mapping and intraoperative neuromonitoring achieved significantly higher rates of GTR than intraoperative techniques alone, with pronounced benefit in eloquent tumors (43.31% vs. 15.09%, *p* = 0.022) [112]. Bello et al. further demonstrated that the integration of preoperative DTI tractography with ISM yielded high sensitivity for identifying critical pathways, including the corticospinal tract (95%) and language tracts (97%), enabling surgeons to anticipate functional boundaries before encountering them [113]. The clinical impact of this multimodal approach is substantial: Staub-Bartelt et al. reported permanent postoperative neurological deficits in only 2% of patients with eloquent-area tumors when comprehensive cortical and subcortical mapping was employed [114], illustrating that aggressive resection can be achieved safely when guided by continuous functional feedback.

Additionally, awake craniotomy allows real-time functional assessment during surgery by integrating behavioral testing with direct cortical and subcortical stimulation. In the GLIOMAP study, Gerritsen et al. performed a propensity score-matched analysis of over 1000 patients with eloquent primary glioblastoma and demonstrated that awake resection was associated with longer median OS (17 vs. 14 months, *p* < 0.001) and lower rates of postoperative neurological deficits compared with surgery under general anesthesia [115]. These findings were reinforced in a meta-analysis by Honeyman et al., which confirmed greater mean EOR, reduced neurological morbidity, and improved OS with awake craniotomy [116]. Importantly, awake approaches have been shown to increase the likelihood of achieving optimal oncologic and functional outcomes with greater NCE resection, lower residual volume, and greater median OS [75]. One study reported GTR without functional loss in 43% of awake cases compared with 26.6% of asleep resections, translating into a median OS of 21.0 vs. 13.0 months [117]. Continuous intraoperative neurophysiologic monitoring further augments this benefit, with Ilgaz Aydinlar et al. demonstrating substantially higher resection volumes and superior postoperative functional scores when multimodal monitoring was used [118]. Collectively, these data establish the essential role of functional mapping, electrophysiologic monitoring and awake surgery as core components of modern glioblastoma surgery that preserve neurological integrity while maximizing oncologic benefit.

### 6.5. Artificial Intelligence and Predictive Modeling

Recent advances in artificial intelligence (AI) have enabled increasingly sophisticated, data-driven approaches to characterizing glioblastoma infiltration beyond the conventional CE border. Multiple groups have demonstrated that deep learning and radiomics applied to preoperative multiparametric MRI can generate recurrence-risk or voxel-wise infiltration maps that reliably identify high-risk NCE tissue that may be amenable to targeting during SMR [119,120,121]. Across independent cohorts, these models have achieved strong predicted performance (OR 8.13–19.48) for predicting regions of future tumor recurrence, albeit with considerable variability in sensitivity (40–97%) and specificity (29–98%) [122]. Complementary efforts have focused on standardizing AI-assisted surgical planning, with automated segmentation frameworks such as the Glioblastoma Surgery Imaging-Reporting and Data System (GSI-RADS) enabling reproducible quantification of tumor volume, spatial distribution and resectability across large multicenter datasets [123,124].

Beyond preoperative planning, AI has also been applied to intraoperative decision-making and tailoring of SMR. Patient-specific mathematical growth modeling has demonstrated that the survival benefit of SMR is strongly modulated by tumor invasiveness, with diffuse tumors deriving greater benefit from extended resection than nodular phenotypes [66]. In parallel, real-time intraoperative AI systems employing optical and fluorescence-based imaging have demonstrated high accuracy for distinguishing tumor from non-tumor tissue at the surgical margin, outperforming expert surgeon judgment [125,126]. Collectively, these studies illustrate how AI is being deployed in the preoperative and intraoperative settings to improve patient outcomes. However, these studies also highlight current limitations of AI models, particularly in predicting postoperative neurological outcomes and integrating these tools into surgical workflows [127].

## 7. Postoperative and Adjunctive Therapeutic Strategies

### 7.1. Residual Infiltrative Disease After Supramaximal Resection

SMR meaningfully reduces the residual burden of infiltrative glioblastoma, yet microscopic disease invariably persists beyond even the most aggressive and safely achievable surgical margins, as evidenced by distant and multifocal recurrences even after aggressive anatomic lobectomy. The remaining infiltrative cells exhibit biological features that confer relative resistance to radiation and systemic therapies. Consequently, while SMR can delay local failure and prolong disease control, it cannot alone eradicate the diffuse cellular reservoirs responsible for recurrence in most patients. Instead, SMR reshapes the postoperative environment, creating a therapeutic window in which adjunctive postoperative strategies may be more effective. In this context, SMR should be conceptualized not as a definitive intervention, but as a foundational strategy that optimizes the conditions under which subsequent local and systemic therapies are applied.

### 7.2. Local and Regional Therapies Targeting the Infiltrative Margin

Several local and regional therapeutic strategies have been developed to address residual glioblastoma following surgical resection. Carmustine (BCNU) wafers, convection-enhanced delivery (CED), and laser interstitial thermal therapy (LITT) represent three distinct approaches that target residual disease. Among these, BCNU wafers remain the most established, with randomized trial data demonstrating modest survival benefit in both newly diagnosed and recurrent glioblastoma [128,129]. However, these trials largely predate the temozolomide-based chemoradiation era, and no randomized evidence supports additive benefit within the contemporary treatment paradigms, limiting current clinical adoption.

CED offers theoretical advantages by bypassing the blood–brain barrier and delivering high local drug concentrations directly into infiltrated tissue, but clinical translation has been constrained by technical challenges related to catheter placement, drug distribution, and treatment durability. While early-phase studies have demonstrated feasibility and biologic activity when adequate tissue coverage is achieved, CED has not consistently demonstrated superiority over standard therapies, highlighting the difficulty of reliably targeting diffuse infiltrative disease [130].

LITT has emerged as a minimally invasive cytoreductive option, particularly for deep-seated or surgically inaccessible tumors [131]. Beyond focal ablation, LITT induces transient blood–brain barrier disruption and local inflammatory responses that may enhance drug delivery and immune engagement, positioning it as a potential platform for combination therapy [132]. Nonetheless, survival outcomes remain variable, complication rates are non-trivial, and optimal patient selection continues to evolve [133].

Collectively, these approaches underscore a unifying principle: reduced tumor burden following SMR may enhance the effectiveness of local therapies, yet no single modality has demonstrated durable benefit across patient populations.

### 7.3. Systemic and Immune-Based Therapies

Systemic and immune-based therapies remain investigational, with no FDA-approved immune-based agents specifically indicated despite extensive clinical trial activity across multiple platforms. Immune checkpoints inhibitors targeting PD-1/PD-L1 and CTLA-4 have demonstrated limited efficacy in glioblastoma, with large randomized trials failing to show benefit in the recurrent setting [134,135,136]. However, emerging data suggest that the timing and route of administration may be critical, as neoadjuvant PD-1 blockade and intracranial dual checkpoint approaches have been associated with enhanced local and systemic immune activation in early-phase studies [137].

Cancer vaccines constitute the largest proportion of glioblastoma immunotherapy trials, with dendritic cell-based and peptide-based platforms demonstrating consistent immunogenicity and encouraging clinical signals [138]. Recent studies combining multivalent DNA vaccines with checkpoint blockade have shown robust vaccine-specific T cell responses, supporting continued investigation of combination strategies and patient selection criteria [139]. CAR T-cell approaches targeting antigens such as EGFRvIII and IL13Rα2 have demonstrated antitumor activity in early clinical trials, with ongoing efforts focused on improving antigen specificity and resistance to tumor heterogeneity [138]. Oncolytic viral therapies represent another promising modality, leveraging direct tumor lysis and immune activation, with several agents advancing through early-phase clinical development.

Across all platforms, efficacy remains constrained by shared challenges including the relative immune privilege of the central nervous system, limited immune infiltration, and restricted drug delivery across the blood–brain barrier [140]. As a result, current efforts increasingly emphasize combination strategies, including integration with radiotherapy, anti-angiogenic agents, metabolic modulation, and novel delivery technologies including focused ultrasound or nanoparticle systems.

### 7.4. Integration of Supramaximal Resection with Postoperative Therapies

While postoperative therapies are central to glioblastoma management, their relationship with SMR has not been well defined. Rather than functioning as independent modalities, surgical cytoreduction and adjuvant therapies should be viewed as complementary components of an integrated treatment strategy. By reducing the burden of residual infiltrative tumor, SMR may enhance the efficacy of downstream therapies through multiple mechanisms. For example, a larger and more uniform resection cavity may improve the distribution of therapeutic agents delivered via convection-enhanced delivery or cavity-directed therapies. Additionally, minimizing residual tumor volume may reduce hypoxic and treatment-resistant niches that currently contribute to the limited effectiveness of radiation and chemotherapy against infiltrative glioblastoma.

From a systemic standpoint, SMR may also influence the tumor immune microenvironment. Glioblastoma is characterized by a profoundly immunosuppressive environment, and reducing the bulk of immunosuppressive signaling may help prime the immune system for improved responses to immunotherapies such as immune checkpoint inhibitors. While these treatments remain under investigation and still require prospective validation, they highlight the potential for SMR to function as a means of optimizing the therapeutic landscape and improving the conditions under which these emerging systemic and immune-based therapies are deployed.

## 8. Challenges, Limitations and Need for Standardization

### 8.1. Strengths and Limitations of This Systematic Review

This study has several important strengths and limitations. First, we performed a comprehensive systematic review across multiple databases, enabling broad capture of the literature evaluating SMR in glioblastoma. Second, given the heterogeneity in how SMR is defined and implemented, we organized the included studies into a structured conceptual framework based on the underlying surgical strategy. This approach allows for more meaningful comparison across studies that would otherwise be difficult to reconcile. Finally, by explicitly addressing sources of bias and limitations in the existing literature, this work aims to provide a balanced and clinically relevant interpretation of the available evidence while offering a reasonable path forward. This systematic review is limited by its reliance of studies with varying designs and definitions of SMR, which precluded quantitative synthesis and meta-analysis. Although a comprehensive search strategy was employed, the possibility of missed studies and publication bias cannot be entirely excluded.

### 8.2. Limitations and Sources of Bias in the Current Evidence

Despite growing enthusiasm for SMR, several conceptual, methodological, and practical limitations continue to constrain its interpretation, generalizability, and widespread clinical adoption. Foremost among these challenges is the absence of a unified definition of SMR. Across the literature, SMR has been variably defined using percentage-based resection of FLAIR abnormality, absolute residual NCE tumor volume, metabolic imaging targets, or anatomic extensions such as lobectomy. While these approaches are individually rational, their heterogeneity complicates cross-study comparison, limits reproducibility, and risks conflating distinct surgical strategies under a single conceptual label. As a result, reported survival benefits may reflect differences in patient selection, tumor biology, or institutional experience rather than a uniform biological effect of extended resection.

Methodological limitations further complicate interpretation of the SMR literature. Most supporting studies are retrospective and originate from high-volume academic centers with advanced imaging, intraoperative mapping, and surgical infrastructure, introducing selection and referral bias. Patients selected for SMR tend to be younger, have higher preoperative functional status, more favorable tumor locations, and greater access to specialized surgical resources, all of which independently influence outcomes. Additionally, confounding by adjuvant therapy and molecular characteristics, including MGMT promoter methylation status, IDH mutation status, and treatment adherence, is inconsistently controlled across studies and may independently impact survival outcomes. Variability in imaging acquisition, segmentation techniques, corticosteroid use, and timing of postoperative imaging further introduces measurement bias into volumetric and percentage-based metrics. In particular, FLAIR abnormalities represent a heterogeneous mixture of infiltrative tumor, edema, and treatment-related changes, and cannot be assumed to uniformly reflect viable disease. These factors underscore the difficulty of using radiographic data alone to define surgical success at the infiltrative margin.

Patient-specific factors further constrain the applicability of SMR. Emerging evidence suggests that the survival benefit of extended cytoreduction is not uniform across all populations and may be attenuated in older patients, those with reduced functional reserve, or tumors involving eloquent or deep structures. Large cohort analyses indicate that while younger, functionally robust patients may derive meaningful benefit from volume-based SMR, these gains diminish in elderly or medically vulnerable populations. Additionally, disparities in awake craniotomy, iMRI, PET imaging, and functional mapping raise concerns regarding treatment access bias, as the technical feasibility of SMR is unevenly distributed across treatment centers.

Neurological safety and functional preservation represent equally critical and often underreported limitations. Although many SMR series report no increase in permanent postoperative neurological deficits, this represents a relatively narrow definition of safety. The true trade-off of more extensive resections, particularly in or near eloquent regions, includes the potential for subtle but clinically meaningful neurocognitive decline affecting language, memory, or executive function. Because postoperative neurological decline negatively impacts tolerance and eligibility for adjuvant therapy, maximal cytoreduction without rigorous functional considerations risks undermining potential survival gains from alternative downstream therapies. Importantly, such outcomes are rarely captured by standard postoperative metrics including KPS or routine neurological examination, introducing outcome reporting bias into the current literature. Emerging studies, such as Leonetti et al., have begun to highlight the importance of formal neurocognitive assessment in the pre- and postoperative care of patients with high-grade gliomas. Because subtle neurological and cognitive decline postoperatively may offset the potential survival benefit of more aggressive cytoreduction, future studies must incorporate standardized evaluation of both oncologic and neurocognitive outcomes to more accurately define the risk–benefit profile of SMR.

The absence of prospective, standardized frameworks for defining EOR and functional outcomes limits cross-study comparability and contributes to uncertainty in interpreting the true impact of SMR. While recent initiatives like the RANO Resect classification represent important progress toward harmonizing volumetric definitions of resection, further validation integrating molecular stratification and functional outcomes is needed. The current body of evidence supporting SMR in glioblastoma is largely derived from retrospective cohort studies, corresponding predominantly to Class III evidence. While several studies demonstrate an association between extended resection and improved survival outcomes, these findings are inherently limited by selection bias, institutional expertise, and variability in SMR definitions. Prospective and randomized data remain limited, and therefore these results should be interpreted with appropriate caution. Until such data emerge, SMR should be regarded not as a surgical mandate, but as a patient-specific intervention that must be applied judiciously within clearly defined boundaries.

## 9. Overall Summary and Future Directions

Synthesizing the available scientific literature on SMR, we propose a pragmatic framework for clinicians, including both surgeons and non-surgeons, to guide its application in glioblastoma. Several key principles emerge from the current literature based upon our review. First, increasing extent of resection is consistently associated with improved survival in glioblastoma. Second, preservation of functional independence is critical, as it directly impacts patient quality of life and the ability to tolerate and complete adjuvant therapies. Third, although multiple adjuncts exist to visualize infiltrative tumor beyond the CE margin, each remains inherently limited in accuracy and reliability. The principle of maximizing tumor resection while preserving function is well established across other oncologic surgical disciplines, such as hepatic resection for colorectal metastases and limb-sparing surgery for sarcoma, and we propose that a similar philosophy should be more deliberately applied to glioblastoma surgery.

In this context, we advocate for a strategy of maximal safe anatomical resection, defined by extension of resection boundaries up to, but not beyond, critical functional structures. In our practice, this includes preservation of key motor and sensory cortex, language regions such as Broca’s and Wernicke’s areas, and key white matter pathways including the corticospinal tracts, somatosensory tract, arcuate fasciculus and corpus callosum. To safely approach these boundaries without violation, we integrate advanced preoperative and intraoperative mapping techniques, including DTI, fMRI, awake language mapping and direct cortical and subcortical stimulation.

This anatomically and functionally grounded approach provides a reproducible framework for SMR that minimizes reliance on imperfect intraoperative tumor visualization adjuncts. By anchoring resection limits to identifiable structural and functional boundaries, surgeons can reduce variability from imaging or metabolic techniques while maximizing cytoreduction.

However, for SMR to be effectively implemented, institutional resources and infrastructure are essential. This includes access to advanced imaging, intraoperative mapping capabilities, and multidisciplinary expertise. Accordingly, we recommend that neurosurgery and neuro-oncology teams evaluate the feasibility and potential benefit of SMR early in the treatment course for each individual patient prior to the initiation of current standard-of-care chemoradiation.

## 10. Conclusions

The emergence of SMR as a biologically and clinically rational extension of maximal safe glioblastoma surgery reflects a growing recognition that infiltrative tumor beyond the CE margin is a principal driver of recurrence and treatment failure. Across diverse frameworks, including FLAIR-based extension, volume minimization, metabolic targeting and aggressive anatomic resections, converging evidence suggests that reducing NCE tumor burden can improve PFS and OS in carefully selected patients. Advances in imaging, intraoperative guidance, and functional mapping have expanded the technical feasibility of SMR in recent years, enabling more aggressive cytoreduction while safely preserving neurological function.

However, SMR should not be regarded as a universal surgical mandate at this time. Its benefits are heterogeneous and appear to be strongly modulated by tumor biology, patient age, functional status, and anatomical constraints. Thus, the future of glioblastoma surgery lies not in resection maximization alone, but in its integration with emerging adjuvant therapies to target residual infiltrative tumors. Within this evolving landscape, SMR represents a powerful, yet selective, tool that must be deployed judiciously as part of an individualized, multidisciplinary approach to glioblastoma care.

## Data Availability

No new data were created in this study. Data sharing is not applicable to this article.

## Supplementary Materials

The following supporting information can be downloaded at: https://www.mdpi.com/article/10.3390/cancers18071182/s1, File S1: Databases.

## Abbreviations

The following abbreviations are used in this manuscript:

5-ALA	5-aminolevulinic acid
AI	Artificial intelligence
ATL	Anterior temporal lobectomy
BCNU	Carmustine
CE	Contrast-enhancing
CED	Convection-enhanced delivery
CEST-EPI	Chemical exchange saturation transfer echo planar imaging
CEUS	Contrast-enhanced ultrasound
DES	Direct electrical stimulation
DiVA	Dual intraoperative visualization approach
DTI	Diffusion tensor imaging
EOFR	Extent of FLAIR resection
EOR	Extent of resection
FDG	18F-Fluorodeoxyglucose
FLAIR	Fluid-attenuated inversion recovery
fMRI	Functional magnetic resonance imaging
GSI-RADS	Glioblastoma surgery imaging-reporting and data system
GTR	Gross total resection
iMRI	Intraoperative magnetic resonance imaging
IOUS	Intraoperative ultrasound
ISM	Intraoperative stimulation mapping
LITT	Laser interstitial thermal therapy
MRI	Magnetic resonance imaging
NCE	Non-contrast-enhancing
OS	Overall survival
PET	Positron emission tomography
PFS	Progression-free survival
PTR	Peri-tumoral resection
RANO	Response Assessment in Neuro-Oncology
SMR	Supramaximal resection
SVZ	Subventricular zone
