# Supplementary material for: Supramaximal Resection in Glioblastoma: Expanding Surgical Boundaries in the Era of Precision Neuro-Oncology—A Systematic Review
Source: Cancers (Basel). 2026 Apr 7;18(7):1182. doi: 10.3390/cancers18071182 (PMC13072266; doi:10.3390/cancers18071182)
Supplement: Supplementary file 1 [file cancers-18-01182-s001.zip › cancers-4212355-supplementary.pdf]

**Supplementary Material**

Search Strings:

Pubmed/MEDLINE: n = 220 reports

((“glioblastoma” [Title/Abstract]) OR (“glioblastoma multiforme” [Title/Abstract]) OR (“GBM” [Title/Abstract])) AND ((“supramaximal resection” [Title/Abstract]) OR (“supramarginal resection” [Title/Abstract]) OR (“supratotal resection” [Title/Abstract]) OR (“extended resection” [Title/Abstract]) OR (“beyond contrast enhancing” [Title/Abstract]) OR (“resection beyond contrast enhancement” [Title/Abstract]) OR (“extent of resection” [Title/Abstract])) AND ((“FLAIR” [Title/Abstract]) OR (“non-contrast-enhancing” [Title/Abstract]) OR (“non contrast enhancing” [Title/Abstract]) OR (“residual tumor volume” [Title/Abstract]) OR (“volumetric resection” [Title/Abstract]) OR (“5-ALA” [Title/Abstract]) OR (“aminolevulinic acid” [Title/Abstract]) OR (“PET” [Title/Abstract]) OR (“fluorescence-guided surgery” [Title/Abstract]) OR (“lobectomy” [Title/Abstract]))

Embase: n = 417 reports

‘glioblastoma’/exp OR ‘glioblastoma’:ti,ab OR ‘glioblastoma multiforme’:ti,ab OR ‘GBM’:ti,ab AND ‘supramaximal resection’:ti,ab OR ‘supramarginal resection’:ti,ab OR ‘supratotal resection’:ti,ab OR ‘extended resection’:ti,ab OR ‘resection beyond contrast enhancement’:ti,ab OR ‘beyond contrast enhancing’:ti,ab OR ‘extent of resection’:ti,ab AND ‘FLAIR’:ti,ab OR ‘fluid attenuated inversion recovery’:ti,ab OR ‘non contrast enhancing’:ti,ab OR ‘residual tumor volume’:ti,ab OR ‘volumetric resection’:ti,ab OR ‘5-ALA’:ti,ab OR ‘aminolevulinic acid’:ti,ab OR ‘fluorescence guided surgery’:ti,ab OR ‘lobectomy’:ti,ab

Web of Science: n = 408 reports

TS=(“glioblastoma” OR “glioblastoma multiforme” OR “GBM”) AND TS=(“supramaximal resection” OR “supramarginal resection” OR “supratotal resection” OR ”extended resection” OR “resection beyond contrast enhancement” OR “beyond contrast enhancing” OR “extent of resection” AND TS=(“5-ALA” OR “aminolevulinic acid” OR “PET” OR “fluorescence-guided surgery” OR “lobectomy”
